# Short‐Term Safety of Nutri‐Jelly in Adults Undergoing Hemodialysis

**DOI:** 10.1002/fsn3.4578

**Published:** 2024-11-12

**Authors:** Janjiraporn Kitiya, Thun Chantaramungkorn, Apinya Pantoe, Chaowanee Chupeerach, Dunyaporn Trachootham

**Affiliations:** ^1^ Master Program in Toxicology and Nutrition for Food Safety, Institute of Nutrition Mahidol University Nakhon Pathom Thailand; ^2^ Hemodialysis Center Rajavej ChiangMai Hospital Chiang Mai Thailand; ^3^ Nutrition Department Rajavej ChiangMai Hospital Chiang Mai Thailand; ^4^ Institute of Nutrition Mahidol University Nakhon Pathom Thailand

**Keywords:** chronic kidney disease, end‐stage renal disease, hemodialysis, malnutrition, Nutri‐jelly, randomized control trial, safety

## Abstract

Excessive water consumption from liquid or reconstituted oral nutrition supplements may increase risk of fluid overload in renal patients. Nutri‐jelly, a ready‐to‐eat texture‐modified diet with 52.8% water, some protein, low potassium, phosphorus, and sodium, could be an alternative. However, its safety is unknown for adults undergoing hemodialysis (HD). This study investigated the short‐term physiological safety of Nutri‐Jelly intake and its preliminary impact on renal outcomes. A randomized open‐label, single‐arm, two‐sequence, two‐period cross‐over trial was conducted in 20 adults undergoing HD with inadequate protein intake (0.50 ‐ 0.70 g/ kg body weight/day). Participants were randomly allocated into 2 groups (*n* =10 each) and assigned in random sequence into both Without‐Jelly (HD 3 times during 7 days) and With‐Jelly periods (100 g Nutri‐Jelly twice daily along with HD 3 times during 7 days). A two‐week washout was between the periods. Outcome measures included adverse symptoms, changes in body weight, heart rate, blood pressure, and blood biochemical parameters relevant to renal outcomes. The results showed no intervention‐related adverse symptoms or significant changes in body weight, heart rate, systolic blood pressure, creatinine, albumin, and sodium. Potassium level and pre‐HD diastolic blood pressure were better controlled during the With Jelly than the Without Jelly Periods (*p* < 0.01 and *p* < 0.05, respectively). The eGFR was improved with no significant difference between the periods. The findings suggest that continuous intake of 100 g Nutri‐Jelly twice daily for 7 days is safe in adults undergoing hemodialysis. Its efficacy on renal‐related parameters warrants further investigations in long‐term studies.

## Graphical abstract

This randomized open‐label, single‐arm, two‐sequence, two‐period crossover trial showed that Nutri‐jelly, a ready‐to‐eat texture‐modified diet with 52.8% water, high protein, low potassium, phosphorus, and sodium, is safe and has good sensory satisfaction in end‐stage renal disease patients undergoing hemodialysis. It could be a new candidate for oral nutrition supplement for patients requiring fluid control.

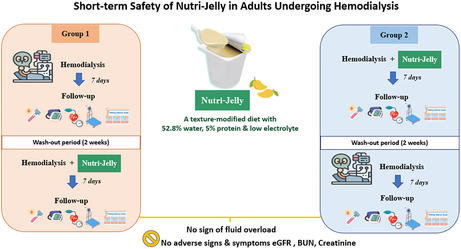

**Abbreviations**
BMI: body mass index
BUN: blood Uurea nitrogen
CKD: chronic kidney disease
ESRD: end‐ stage renal disease
GFR: glomerular filtration rate
HD: hemodialysis
IDW: ideal body weight
*N*
: number of participants

## 1 Introduction

End‐stage renal disease (ESRD) is defined as a glomerular filtration rate (GFR) < 15 mL/min/1.73 m^2^ (Sgambat et al. 2019). It is the terminal and irreversible stage of chronic kidney disease (Gusev et al. 2021). The prevalence of ESRD in East and Southeast Asia has increased, while those in most high‐income countries remain unchanged (Thurlow et al. 2021). Factors contributing to the rise in ESRD include changes in lifestyle, dietary habits, and underlying diseases such as diabetes mellitus and hypertension. Treatment of ESRD includes hemodialysis and renal transplant. Hemodialysis is the major therapy, which can help filter the blood to remove waste (Thurlow et al. 2021). Several studies reported significantly improved survival after hemodialysis compared with conservative management (Chandna et al. 2011; O'Connor and Kumar 2012). Without hemodialysis, ESRD patients may accumulate toxic substances in the body and have fluid and electrolyte imbalance which leads to heart failure and death. A retrospective study reported that ESRD patients survived for only 7.4 days after the discontinuation of dialysis (O'Connor et al. 2013).

Malnutrition was found in 45% of ESRD patients undergoing hemodialysis (Badrasawi et al. 2021; Sabatino et al. 2018; Gluba‐Brzózka, Franczyk, and Rysz 2017). The major causes of malnutrition are dialysis‐derived loss of amino acids, and inadequate protein and energy intake (Gluba‐Brzózka et al. 2020; Sabatino et al. 2018; Carrero and Cozzolino 2014). Protein energy wasting (PEW), a gradual decline in body stores of protein and energy, is found in 30%–70% of these patients (Fouque et al. 2008; Sarav and Kovesdy 2018). PEW is strongly linked to poor clinical outcomes, an elevated rate of hospitalization, complications, and death (Badrasawi et al. 2021; Sgambat et al. 2019; Gluba‐Brzózka, Franczyk, and Rysz 2017; Kim et al. 2013). Thus, the National Kidney Foundation Kidney Disease Outcomes Quality Initiative and the Academy of Nutrition and Dietetics (KDOQI/AND) 2020 recommend dialysis patients consume 25–35 kcal of energy/kg body weight/day, and 1.0–1.2 g protein/ kg body weight/day (Ikizler et al. 2020). Unfortunately, most patients' dietary intakes still cannot meet the requirements (Sahathevan et al. 2020; Hendriks, Kooman, and van Loon 2021). About 50% of the patients consumed < 1.0 g protein/kg body weight/day (Yang et al. 2020; Hendriks, Kooman, and van Loon 2021).

To reduce the risk of malnutrition, oral nutrition supplements (ONS) with high protein and energy are available and were shown to improve biomarkers of nutrition (serum albumin and mid‐arm muscle circumference) in ESRD patients with hemodialysis (Mah et al. 2020; Liu et al. 2018). The ideal oral nutritional supplement for this specific group of patients should contain a higher energy and protein density and lower phosphate, potassium, and sodium contents, compared with the conventional formula (Sabatino et al. 2018). However, low compliance with oral nutritional supplementation is often observed in hemodialysis patients due to inconvenience in preparation and low sensory satisfaction (Hendriks, Kooman, and van Loon 2021; Williams and Summers 2009). Most available ONS are ready‐to‐eat liquid or powder to be freshly dissolved into a liquid. Unfortunately, patients and caregivers often forget that the liquid from food must also be counted as fluid intake. Therefore, they may drink additional water, and fluid overload may occur even when consuming ready‐to‐eat liquid food. For the powder form, incorrect preparation of ONS, for example, wrong dilution or mixing can lead to excessive water consumption but low nutrient intake (Piccoli et al. 2020; Banerjee, Rosano, and Herzog 2021). Consequently, fluid overload is a common complication for ESRD patients, which could lead to heart failure and death (Banerjee, Rosano, and Herzog 2021; Loutradis et al. 2021). Therefore, a ready‐to‐eat, nutrient‐dense, low water‐containing, and sensory‐acceptable diet is warranted as an alternative for patients undergoing hemodialysis.

Nutri‐Jelly is a ready‐to‐eat nutritious texture‐modified diet. The basic structure is a milk‐derived colloid. It has a semisolid texture that can be melted in the mouth and is easy to swallow. One serving (100 g) of Nutri‐jelly provides 130 Kcal energy, 5 g of protein, 175 mg of potassium, 86.8 mg of phosphorus, 46.9 mg of sodium, and 52.8 g of water. The amount of water in Nutri‐Jelly is 47.2% less than that of commonly available liquid ONS (1:1, 100 g contains 100 g water).

Previous studies showed that Nutri‐jelly improves the quality of life and decreases tube feeding demand in head and neck cancer patients (Trachootham et al. 2015). Based on moderate water content and nutrient profile, Nutri‐jelly may be a good candidate oral nutritional supplement for hemodialysis patients. Nevertheless, the safety of Nutri‐jelly in this group of patients was unknown. In this study, this randomized, single‐blinded, crossover‐controlled trial was aimed to investigate the short‐term safety of Nutri‐Jelly in end‐stage renal disease (ESRD) patients undergoing hemodialysis. Adverse symptoms, body weight, and renal‐related blood parameters were monitored in the 7‐day periods of hemodialysis with or without Nutri‐Jelly intake. Sensory satisfaction of the product was also evaluated.

## 2 Materials and Methods

### 2.1 Materials

**FIGURE 1:**
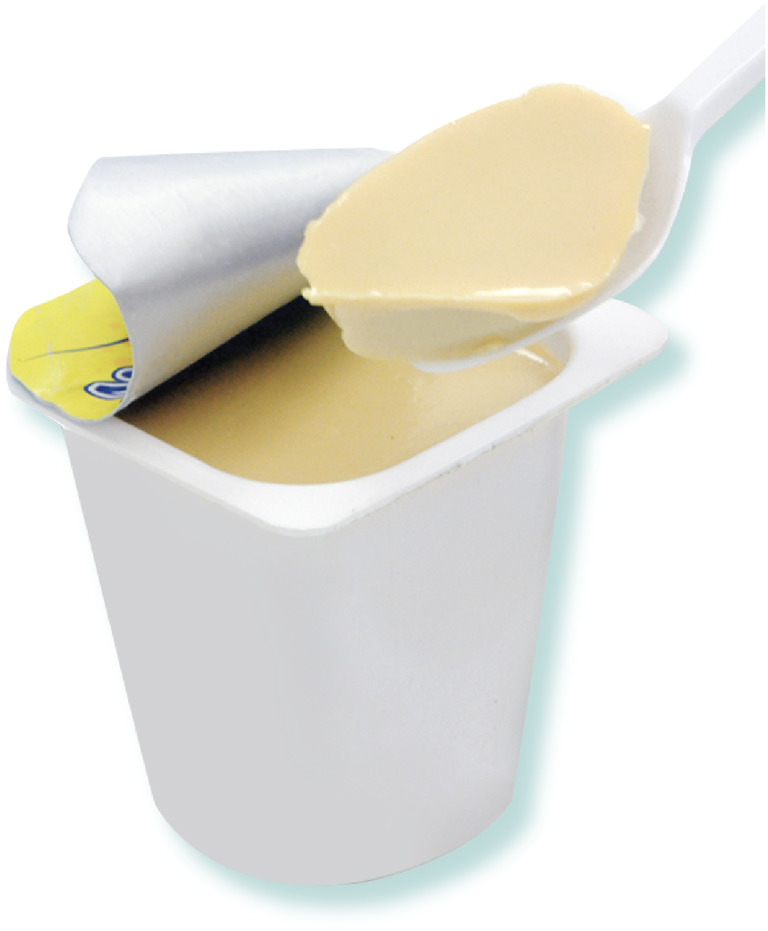
Nutri‐jelly mango‐flavored. It has a semisolid texture at International Dysphagia Diet Standardization Initiative (IDDSI) level 5 under room temperature and level 4 under 37°C.

Nutri‐jelly (mango‐flavored) (Figure 1) was obtained from the Dental Innovation Foundation under Royal Patronage, His Majesty the King's Dental Service Unit. It was produced by ultra‐high temperature (UHT) processing and packed with aseptic filling into a 100 g cup. It was produced under international standards (Good Manufacturing Practice (GMP), Hazard Analysis and Critical Control Points (HACCP), and International Organization for Standardization (ISO) 22,000). The shelf‐life under room temperature was 8 months. The product was HALAL‐certified and approved by the Thai Food and Drug Administration with registration number 10‐1‐09760‐5‐0002.

### 2.2 Study Design

A randomized open‐label, single‐arm, two‐sequence, two‐period crossover trial was conducted at Hemodialysis Center, Rajavej Chiang Mai Hospital, Chiang Mai, Thailand. All participants received Nutri‐Jelly. Participants were randomly allocated into two groups (*n* = 10 each). The randomization with minimization was performed by matching gender, age, HbA1C, body mass index (BMI), ideal body weight (IDW), and historical time of hemodialysis. Both groups were assigned in random sequence into both without‐jelly (hemodialysis three times for a week) and with‐jelly periods (100 g Nutri‐jelly twice daily along with hemodialysis three times for a week). A 2‐week washout was between the periods. Group 1 was first assigned to the without‐jelly period followed by the with‐jelly period. Group 2 was first assigned to the with‐jelly period followed by the without‐jelly period. The data were analyzed to compare the outcomes between the with‐jelly and without‐jelly periods of the same individual. Though participants were aware of their without‐jelly and with‐jelly periods, the laboratory analyzers and the statistical analyzers were concealed from the random assignment throughout the study.

### 2.3 Ethical Consideration

This study was conducted at the Institute of Nutrition, Mahidol University. The study protocol was approved by the Mahidol University Central Institutional Review Board (COA. No. MU‐CIRB 2023/273.1110). It was performed according to the Declaration of Helsinki and was registered at the Thai Clinical Trial Registry (TCTR20230507001). Informed written consent was obtained from each participant before the study. The protocol of the Thai clinical trials registry can be accessed at https://www.thaiclinicaltrials.org/show/TCTR20230507001.

### 2.4 Sample Size and Power

Since there were no studies on the effects of Nutri‐jelly on CKD patients, the sample size was calculated by using G Power 3.1.9.4 with a theoretical large effect size of 0.8, and the power value of 0.8 for the paired *t*‐test. The required sample size is at least 15. With a 30% dropout rate, 20 participants were recruited.

### 2.5 Participants

Inclusion criteria were 18–65 years old, diagnosed with ESRD, undergoing hemodialysis three times a week for at least 1 year, outpatients, HbA1c < 9, acceptable to eat Nutri‐jelly, serum albumin of 2.0–4.0 g/dL, plasma potassium of < 6 mg/dL, dry ideal body weight (IBW) of 50–60 kg, protein intake of 0.50–0.70 g/ kg body weight/day (a daily protein intake of 25–42 g per day). Since Nutri‐jelly is intended to be used as an oral nutrition supplement, especially for those consuming inadequate protein, the baseline protein intake at 0.5–0.7 g protein/kg body weight/day was set to represent the target group of the product, and to minimize variability in baseline nutritional status. Correspondingly, two cups of Nutri‐Jelly (giving an additional 10 g protein/ day) was assigned to meet the optimum protein requirement for this group of patients. The exclusion criteria included having changes in medicine used, active infection, active malignancy, heart failure, pregnancy, allergic to animal food sources (milk, egg, fish, and shrimp), routine consumption of other oral nutritional supplements, personal use of dietary supplements (not prescribed or recommended by physicians), being unable to make a reliable decision or effective communication. All participants signed their written informed consent before data collection. Their identities were protected, following the International Conference on Harmonization Good Clinical Practice (ICH‐GCP).

### 2.6 Intervention and Study Procedure

Nutri‐jelly is composed of 52.8% water, 21.8% finely minced mango, 10% milk powder, 5.5% sugar, 2.4% whey protein, 0.95% rice bran oil, sugar substitute (INS 420), acidity regulator (INS 330), Stabilizers (gelatin), INS 406 (agar), and INS 415 (xanthan gum). As shown in Table S1, A cup of Nutri‐jelly (100 g) provides 130 kcal of energy, 5 g of protein, 18 g of carbohydrate, 52.8 g of water, 175 mg of potassium, 86.8 mg of phosphorus, and 46.9 mg of sodium. This study used a randomized open‐label, single‐arm, two‐sequence, two‐period crossover design with two assigned periods, that is, without‐jelly and with‐jelly periods. A 2‐week washout was between the periods. During the without‐jelly period, all participants received hemodialysis three times during 7 days, for example, Monday, Wednesday, and Friday. They were instructed to maintain their usual diet. During the with‐jelly period, all participants consumed 100 g Nutri‐Jelly twice daily and hemodialysis three times during 7 days. On the days without dialysis, a cup was consumed each meal for two meals (a total of 200 g per day). On the days with dialysis, two cups were taken consecutively after dialysis (a total of 200 g per day). Over the 7 days, each participant received a total of 1400 g Nutri‐jelly. Throughout the study, all participants daily wrote their Nutri‐jelly intake (for compliance check) and any adverse symptoms in their subject diaries. They also made 24‐h dietary records daily of other food they consumed. They were asked to avoid taking other oral supplement products.

### 2.7 Outcome Measurement

Safety parameters including adverse symptoms, such as nausea, vomiting, diarrhea, itching, fatigue, and edema, were monitored daily by the participants and recorded in subject diaries. Dietitians and nurses checked the diet records and diaries every time the patients visited the hospital for dialysis (three times per week). Other outcome parameters were measured at baseline and after 7 days during the without‐jelly and with‐jelly periods. Total body weight was determined using a weighing and BMI scale, NAGATA model BW‐160 SERIES (Thai Metrology Group CO., LTD., products of Taiwan). Blood pressure and pulse rate were measured using a blood pressure monitor OMRON model HBP‐1300 (OMRON Healthcare Co., Ltd., Kyoto, Japan). In each hemodialysis session, blood pressure was measured twice, that is, at predialysis and postdialysis. Three repeated measurements were performed to identify the average. Blood biochemistry (glomerular filtration rate [GFR], blood urea nitrogen [BUN], creatinine [CR], albumin, potassium, phosphorus, and sodium) was measured weekly using a Clinical Chemistry Analyzer, VITROS Model 7600 Dry Chemistry Principal Ortho Clinical Diagnostics Company USA. Signs of fluid overload (hypervolemia) were monitored for edema, weight gain, high blood pressure, and increased blood sodium (Loutradis et al. 2021).

### 2.8 Satisfaction

Patients' sensory satisfaction for Nutri‐jelly was evaluated by using 5‐point hedonic scale where participants rated their satisfaction across multiple dimensions. These dimensions included appearance, color, smell, taste, texture, and overall liking of the product. The scale was defined as follows: a score of 5 indicated “Very Satisfied,” 4 corresponded to “Satisfied,” 3 to “Neutral,” 2 to “Dissatisfied,” and 1 to “Very Dissatisfied.”

### 2.9 Statistical Analysis

Numerical and categorical data were summarized as mean value ± standard deviation and frequencies (in %), respectively. Normality was checked by using the Shapiro–Wilk test before selecting statistical tests. Baseline characteristics between the G1 group and the G2 group were compared using Fisher's exact and chi‐square tests for categorical data, and unpaired *t*‐tests for numerical data. Changes in each parameter over time within the same group were analyzed by using the Wilcoxon matched‐pairs signed rank test. A comparison of parameter changes over time between dialysis with Nutri‐jelly and dialysis without‐jelly periods was made by using a mixed‐effects model (time × group), followed by the Sidak test. A *p* value of < 0.05 was considered statistically significant. Graphing and statistical analysis were performed by using GraphPad Prism V 10.0.0.

## 3 Results

### 3.1 Participants, Compliance, and Adverse Symptoms

**FIGURE 2:**
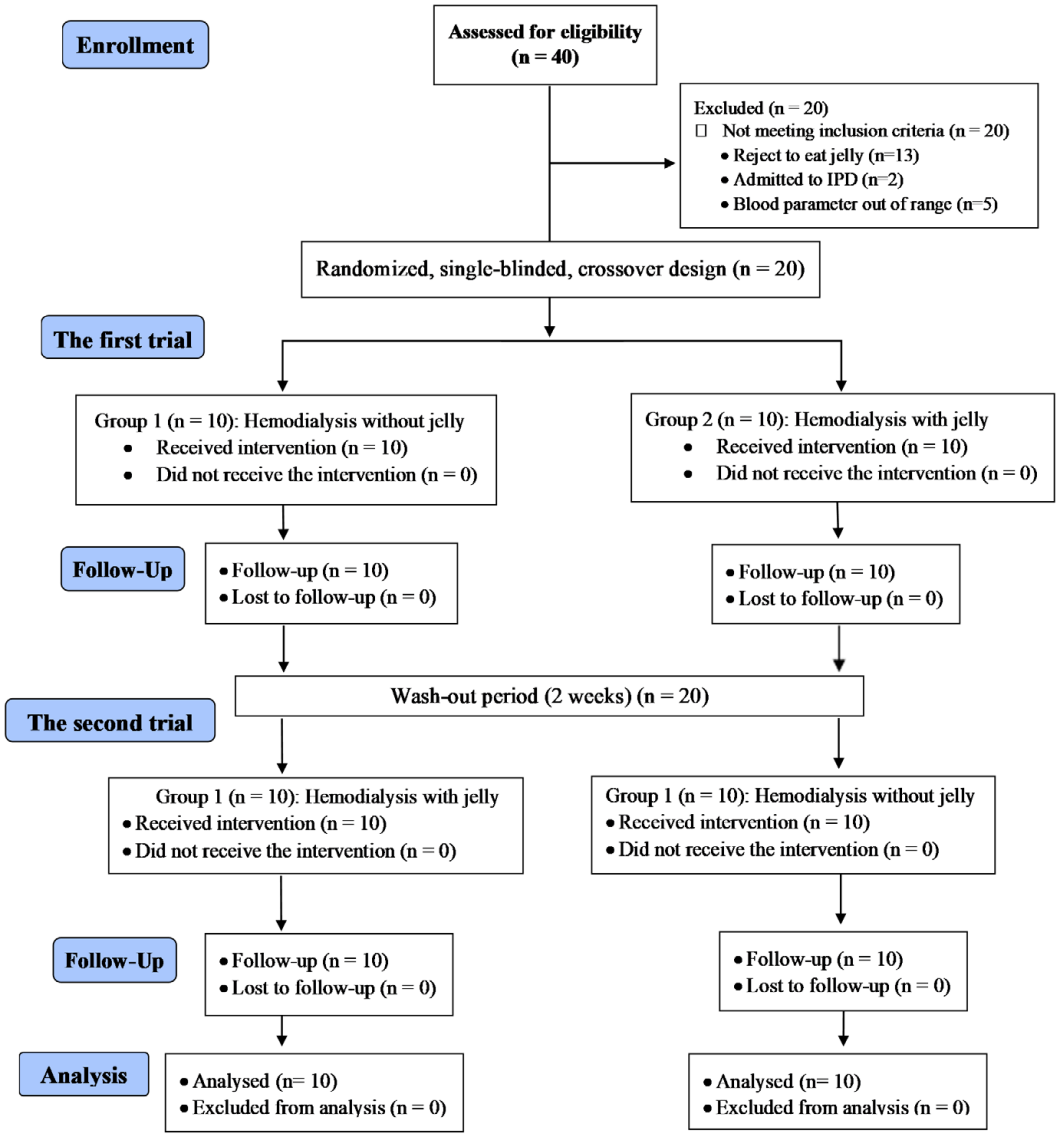
Consolidated Standards of Reporting Trials (CONSORT) participant flow diagram. The number of recruited, randomized, and analyzed participants is shown.

The data were collected from January–March 2023. As shown in Figure 2, 40 participants were initially screened and 20 of them passed. Then, they were randomly allocated into two groups (Group 1: G1, Group 2: G2, *N* = 10 each). Participants in G1 received dialysis without jelly in the first trial followed by Nutri‐jelly in the second trial. Participants in G2 received dialysis with jelly first followed by dialysis only without jelly in the second trial. A 7‐day washout period was between both trials. The washout period was included to ensure that any residual effects of the Nutri‐jelly or others from the previous phase did not carry over into the next phase and ensure that the results accurately reflect the impact of the Nutri‐jelly. All participants in both groups adhered to protocol and came for follow‐up with no dropout. Thus, intention‐to‐treat data analysis was used.

As shown in Tables 1 and 2, gender, education, occupation, time to hemodialysis, age, weight, HbA1C, BMI, IDW, and baseline blood biochemical parameters in both groups are not significantly different. All participants (*n* = 20) finished two cups a day of Nutri‐jelly according to the protocol (Table S2). No adverse symptoms were observed either without jelly or with jelly during the study (Table S3). Edema, the major symptom of fluid overload was not found throughout the study.

**TABLE 1:** Demographic characteristics of the participants at baseline.

Parameter	G1 (*N* = 10)	G2 (*N* = 10)	*p*
	*N* (%)	*N* (%)	
Gender			
Male	4 (40)	4 (40)	> 0.9999*
Female	6 (60)	6 (60)	
Education			
Primary school	3 (30)	4 (40)	0.16**
Secondary school	0 (0)	3 (30)	
College	1 (10)	1 (10)	
University	6 (60)	2 (20)	
Occupation			
Agriculture	1 (10)	0 (0)	0.499**
Employee	4 (40)	2 (20)	
Unemployed	2 (20)	3 (30)	
Retailer	3 (30)	5 (50)	
Time to hemodialysis (years)			
1–4 years	5 (50)	4 (40)	0.99*
5 years and above	5 (50)	6 (60)	

	Mean ± SD	Mean ± SD	*p* value***
Age (years)	46.80 ± 9.27	47.70 ± 11.98	0.85
Weight (kg)	65.95 ± 14.68	62.36 ± 13.11	0.57
BMI (kg/m^2^)	24.98 ± 4.33	23.32 ± 3.15	0.33
IDW (kg)	59.00 ± 10.77	59.90 ± 12.30	0.86

Abbreviations: BMI, body mass index; G1, group 1 (without jelly before jelly) and G2, group 2 (with jelly before without jelly); IDW, Ideal body weight; *N*, the number of participants.

*p* values were obtained from *Fisher's exact test; **Chi‐square test; ***Unpaired *t*‐test.

**TABLE 2:** Baseline blood biochemical parameters.

Parameter	G1 (*N* = 10)	G2 (*N* = 10)	*p*
	Mean ± SD	Mean ± SD	
GFR (mL/min/1.73 m^2^)	4.63 ± 1.99	3.46 ± 0.73	0.08*
BUN (mg/dL)	61.50 ± 15.54	62.80 ± 11.38	0.83**
Creatinine (mg/dL)	10.61 ± 2.73	12.92 ± 3.22	0.10**
Albumin (mg/dL)	3.88 ± 0.49	3.74 ± 0.39	0.49**
Potassium (mg/dL)	4.11 ± 0.46	4.48 ± 0.88	0.25**
Phosphorus (mg/dL)	5.12 ± 2.46	5.16 ± 2.46	0.62**
Sodium (mg/dL)	137.4 ± 1.95	138.3 ± 3.16	0.45**
HbA1C (%)	5.73 ± 0.89	5.68 ± 0.63	0.54*

Abbreviations: BMI, body mass index; BUN, blood urea nitrogen; GFR, glomerular filtration rate; HbA1C, hemoglobin A1C; *N*, the number of participants.

*p* values were obtained from *Mann–Whitney test; **Unpaired *t*‐test.

### 3.2 Effect of Nutri‐Jelly on Body Weight, Heart Rate, Blood Pressure, and Kidney Function

**FIGURE 3:**
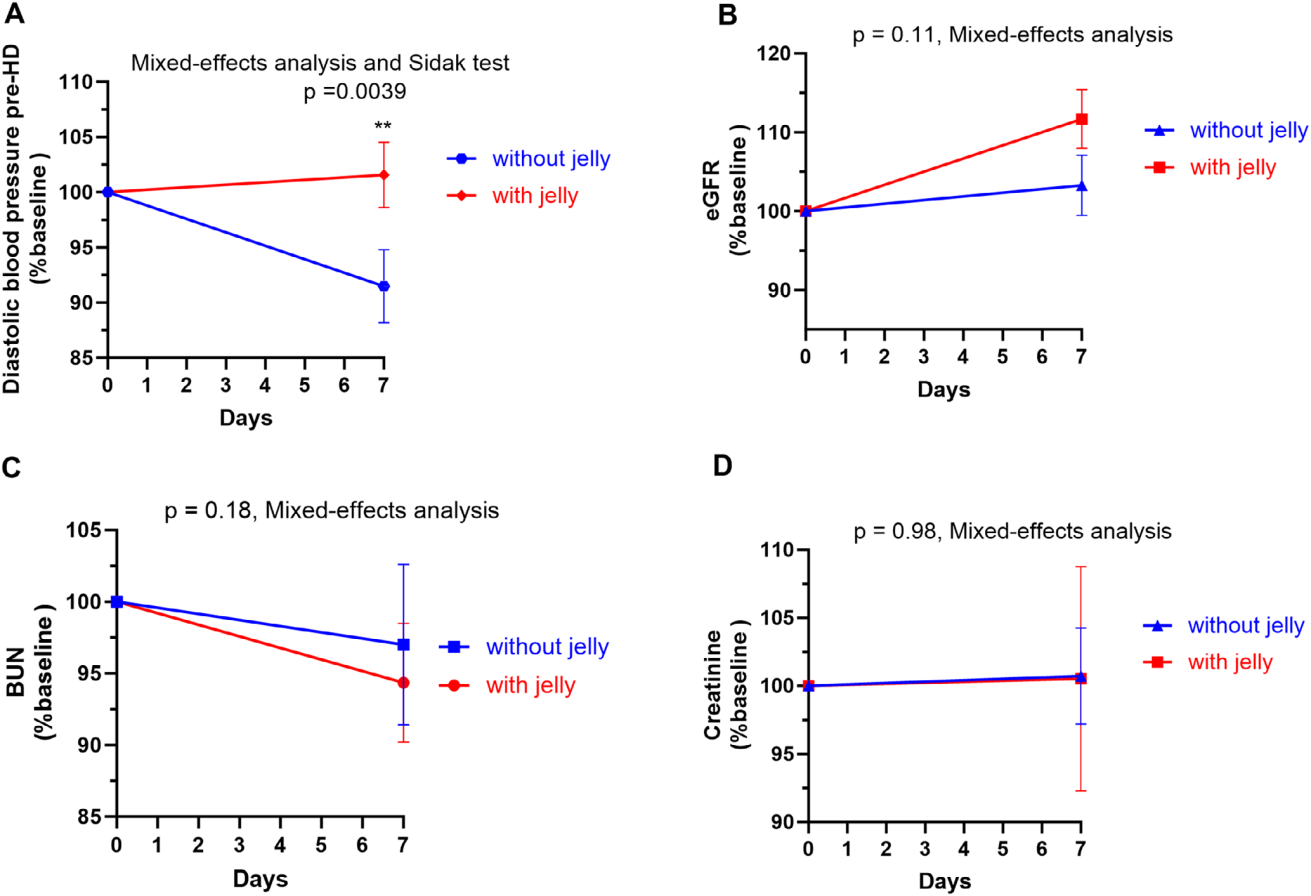
Comparison of prehemodialysis (pre‐HD) diastolic blood pressure and blood biomarkers of kidney function during the with‐jelly and without‐jelly period. The line graph shows the mean and standard deviation for % baseline values of pre‐HD) diastolic blood pressure (A), eGFR (B), BUN (C), or creatinine (D) in the with‐jelly (red) and without‐jelly (blue) periods. *p* values were obtained from mixed effect analyses followed by Sidak's multiple comparison tests. (Comparison between with vs. without jelly over time). **Represents *p* < 0.01.

As shown in Table 3, pre‐ and posthemodialysis (pre‐HD and post‐HD) body weight, heart rate, systolic blood pressure, and post‐HD diastolic blood pressure were not significantly changed during with and without jelly periods. While the average prehemodialysis (pre‐HD) diastolic blood pressure was dropped in the without‐jelly period (*p* = 0.008), there was no change in the with‐jelly period (*p* = 0.83). Figure 3A shows that the pre‐HD diastolic blood pressure was significantly better maintained during the with‐jelly period than the without‐jelly period (*p* = 0.0039). Weight gain and increased blood pressure, the signs of fluid overload, were not found throughout the study. Table 4 shows that the average estimated glomerular filtration rate (eGFR) is significantly increased after consuming Nutri‐jelly (*p* = 0.01). However, the change was not significantly different from the without a jelly period (Figure 3B, *p* = 0.11). Blood urea nitrogen (BUN) and creatinine (CR) were not significantly altered in both periods (Table 4, Figure 3C,D).

**TABLE 3:** Changes in body weight, heart rate, and blood pressure.

Parameter	Without jelly (*N* = 20)		*p*	With jelly (*N* = 20)		*p*
	mean ± SD			mean ± SD		
	0 Day	7 Days		0 Day	7 Days	
Pre‐HD weight (kg)	65.71 ± 13.87	64.97 ± 13.57	0.74	66.78 ± 14.42	66.57 ± 14.31	0.21
Post‐HD weight (kg)	64.41 ± 13.58	64.35 ± 13.61	0.06	66.77 ± 13.40	66.12 ± 13.44	0.65
Heart rate pre‐HD (Pulse/min)	83.20 ± 10.75	84.85 ± 14.90	0.66	83.85 ± 14.74	81.55 ± 13.99	0.45
Heart rate post‐HD (Pulse/min)	77.95 ± 14.20	74.85 ± 12.83	0.44	76.80 ± 12.30	77.45 ± 11.06	0.96
Systolic blood pressure pre‐HD (mmHg)	156.05 ± 16.71	155.00 ± 18.09	0.95	149.35 ± 17.56	148.90 ± 13.89	0.99
Diastolic blood pressure pre‐HD (mmHg)	86.45 ± 14.64	78.4 ± 15.50	0.008**	78.25 ± 13.57	79.65 ± 17.72	0.83
Systolic blood pressure post‐HD (mmHg)	152.35 ± 19.07	151.85 ± 18.45	0.99	152.55 ± 14.83	149.4 ± 18.61	0.76
Diastolic blood pressure post‐HD (mmHg)	83.15 ± 14.40	80.4 ± 13.93	0.52	82.00 ± 11.54	78.60 ± 13.76	0.38

Abbreviations: BMI, body mass index; BUN, blood urea nitrogen; GFR, glomerular filtration rate; HbA1C, hemoglobin A1C; *N*, the number of participants.

*p* values were obtained from mixed effect analyses followed by Sidak test (Comparison between 0 and 7 days of each period).

** Represents *p* < 0.01.

**TABLE 4:** Comparison of blood biochemical parameters before and after each intervention's periods.

Parameter	Without jelly (*N* = 20)		*p*	With jelly (*N* = 20)		*p*
	Mean ± SD			Mean ± SD		
	0 Day	7 Days		0 Day	7 Days	
eGFR (mL/min/1.73 m^2^)	4.13 ± 1.53	4.23 ± 1.58	0.75	4.08 ± 1.43	4.50 ± 1.48	0.0107 *
BUN (mg/dL)	68.90 ± 10.79	66.30 ± 17.73	0.68	67.45 ± 15.70	62.35 ± 14.12	0.25
Creatinine (mg/dL)	11.72 ± 3.02	11.74 ± 3.46	0.99	11.62 ± 3.56	11.03 ± 3.11	0.28
Albumin (mg/dL)	4.06 ± 0.42	4.04 ± 0.44	0.90	4.11 ± 0.43	4.05 ± 0.44	0.49
Potassium (mg/dL)	4.34 ± 0.67	4.57 ± 0.65	0.11	4.44 ± 0.68	4.36 ± 0.64	0.73
Phosphorus (mg/dL)	6.03 ± 2.60	5.90 ± 2.51	0.29	5.73 ± 2.26	5.53 ± 1.88	0.37
Sodium (mg/dL)	138.5 ± 2.18	137.8 ± 1.60	0.70	138.1 ± 1.80	137.85 ± 2.43	0.25

*Note:* This table shows the mean ± SD of each characteristic as specified.

Abbreviations: BUN, blood urea nitrogen; GFR, glomerular filtration rate; *N*, the number of participants.

*p* values were obtained from mixed‐effects analysis followed by Sidak's test.

* Represents *p* < 0.05.

### 3.3 Effect of Nutri‐Jelly on Blood Levels of Albumin, Sodium, Potassium, Phosphorus, and Calcium

**FIGURE 4:**
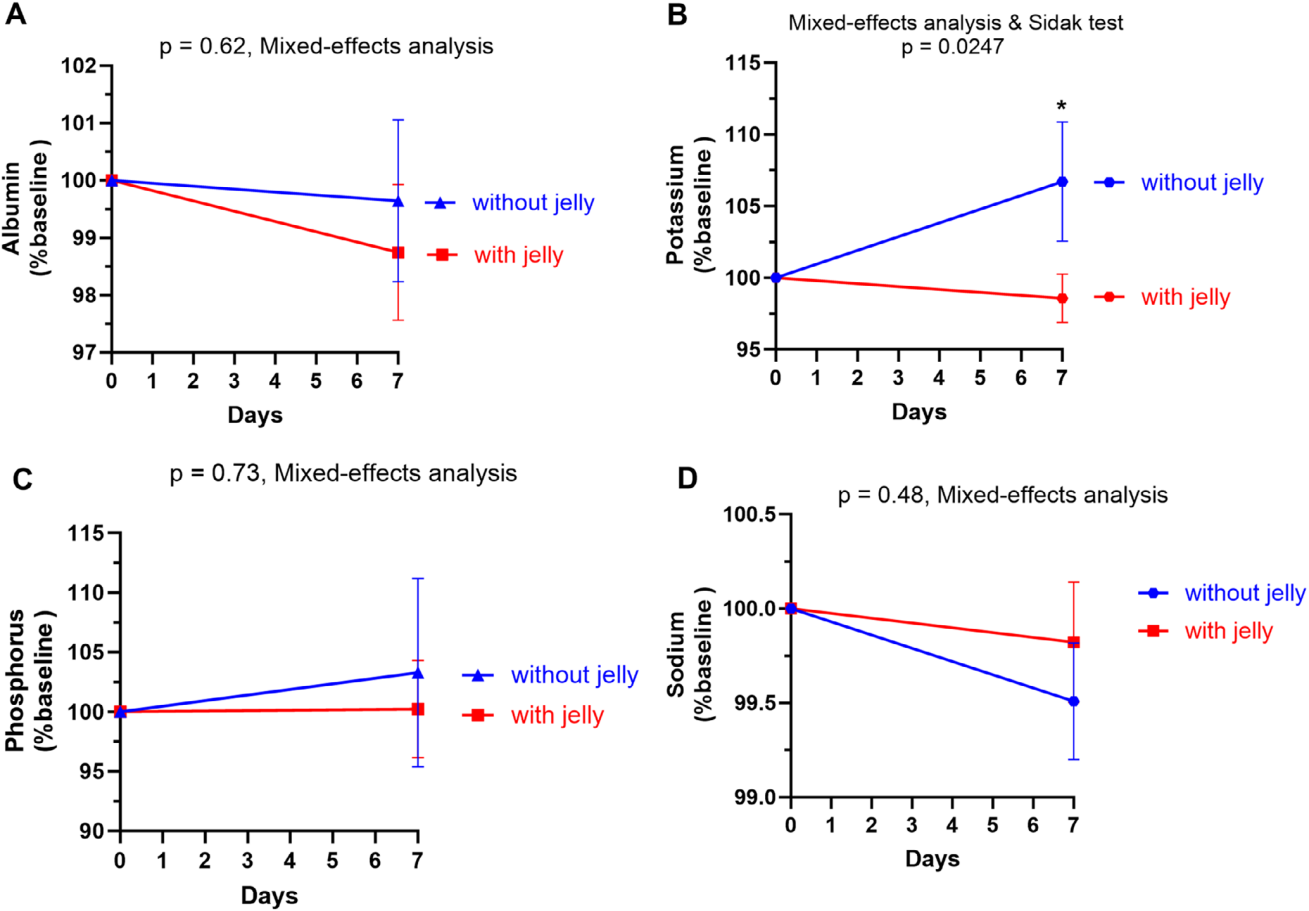
Changes in blood levels of albumin and electrolytes during with‐jelly and without‐jelly periods. The line graph shows the mean and standard deviation of albumin (% baseline) (A), potassium (B), phosphorus (C), or sodium (D) in the with‐jelly (red) and without‐jelly (blue) groups. *p* values were obtained from mixed effect analyses followed by Sidak's multiple comparison tests. (Comparison between with vs. without jelly over time). *Means *p* < 0.05.

The average albumin levels are not significantly altered in both periods (Table 4). Though the albumin after Nutri‐jelly intake is slightly lower than that of the without‐jelly period, the difference between groups is not significant (Figure 4A, *p* = 0.62). Interestingly, the % baseline of potassium level during the with‐jelly period is significantly lower than that of the without‐jelly period (Figure 4B, *p* = 0.02). The average phosphorus and sodium during with‐ and without‐jelly periods are slightly reduced with no significant difference between periods (Table 4, Figure 4C,D **)**. Increased blood sodium, a common sign of fluid overload was not found throughout the study.

### 3.4 Changes in Energy and Nutrient Intakes During the Study

The average nutrient and energy intakes of other food besides Nutri‐jelly on hemodialysis and nonhemodialysis days during the with‐jelly period are not significantly different from those of without‐jelly periods (Table S1).

### 3.5 Satisfaction

The participants expressed satisfaction with Nutri‐jelly. The average scores for each attribute were as follows: An average score of 3.85 for appearance, 3.95 for color, and 4 for smell, taste, and texture. The overall liking of Nutri‐Jelly was rated at 4.05. Ninety‐five percent of participants rated Nutri‐jelly with an overall score of 3–5, suggesting that most ESRD patients with hemodialysis are satisfied with Nutri‐jelly.

## 4 Discussion

Oral protein‐based nutrition supplements were proven useful in improving nutritional status in ESRD patients undergoing hemodialysis (Hendriks, Kooman, and van Loon 2021; Mah et al. 2020). Unfortunately, the currently available supplements for dialysis patients are not well‐received with up to 35% of patients disliking the taste products (Williams and Summers 2009). Furthermore, most complete nutrition formulas are liquid supplements or powders for dissolving in water. So, it is quite challenging to control fluid intake and prevent fluid overload (Piccoli et al. 2020). In this study, we proposed Nutri‐jelly, a texture‐modified diet with 58% water content as an alternative nutritional supplement for ESRD patients on hemodialysis. This randomized open‐label, single‐arm, two‐sequence, two‐period crossover control trial demonstrated that an intake of two cups per day of Nutri‐jelly (total of 200 g per day) for 7 days is safe in hemodialysis patients. No adverse signs and symptoms and no signs of fluid overload were observed. Furthermore, there is a positive trend in increasing eGFR after Nutri‐jelly intake, compared to baseline. Also, during the period of Nutri‐jelly intake, blood potassium levels and pre‐hemodialysis diastolic blood pressure are better controlled than those of the without‐jelly period. Importantly, the overall sensory satisfaction of Nutri‐jelly is good. Though the protein content in Nutri‐jelly is not as high as most commercial ONS, the low water‐containing advantage and the good sensory acceptance make it a choice to consider when fluid control is the main concern. Future research and development to increase protein content in Nutri‐jelly without increasing water content is encouraged.

The improvement in eGFR and better control of potassium and diastolic pressure could be explained by two possible mechanisms. One is the change in dietary intake. Adding two cups of Nutri‐jelly gives an additional energy of 260 kcal. The patients may feel full and consumption of other food with high loading of electrolytes may be lessened. Compared with regular protein supplements, Nutri‐jelly supplementation has the advantage of providing both protein and energy. Usually, inadequate energy intake will lead to muscle protein degradation and the rise of serum creatinine, resulting in a reduction in eGFR value. Protein supplement also increases serum creatinine levels (Butani et al. 2002), which may worsen the eGFR. In contrast, Nutri‐jelly provides both energy and protein, which may help prevent muscle degradation and creatinine rise, and consequently improve the eGFR value. Another possibility is the anti‐inflammatory effects of Nutri‐jelly. Nutri‐jelly is made from mango puree (21.8 g of mango in 100 g Nutri‐jelly), which may contain some phytochemicals such as carotenoids and mangiferin which could provide anti‐inflammatory effects (Zarasvand et al. 2023; Kabir, Shekhar, and Sidhu 2017). Since the pathogenesis of kidney disease involves chronic inflammation (Kadatane et al. 2023), the anti‐inflammatory effects of Nutri‐jelly in ESRD patients warrant further studies. Future clinical trials should measure changes in inflammatory cytokines such as high‐sensitivity C‐reactive protein (hs‐CRP) (Lestariningsih et al. 2019).

Previous studies suggest that sensory disliking and inconvenience in preparation are one of the main reasons dialysis patients refuse to consume oral nutritional supplements (Hendriks, Kooman, and van Loon 2021). The satisfaction of the existing popular branded supplements by hemodialysis patients is not good evidenced by 25%–35% of patients disliking the renal‐specific oral nutritional supplements products (Williams and Summers 2009). Interestingly, in this short‐term study, all patients accepted Nutri‐jelly well with 95% overall satisfaction. Most participants were satisfied with the odor and taste. However, they were less satisfied with the appearance and color of the jelly. To improve the acceptability of Nutri‐jelly by hemodialysis patients, modifications in the color and appearance of the jelly may be considered. Owing to the good sensory acceptance, moderate water content, and safety reported in this work, Nutri‐jelly could be a good candidate for an oral nutritional supplement for ESRD patients undergoing hemodialysis deserving further exploration.

This research has several strengths. First, the randomized crossover design allows the comparison of parameters between with‐ and without‐jelly periods in the same individuals. Thus, the analysis of data with individual variation becomes more reliable. Furthermore, this study has perfect compliance with the complete consumption of Nutri‐jelly and follow‐up according to the protocol. No subjects have dropped out during the study. Nevertheless, there was some limitation. First, it is an open‐label study. Participants received no interventions during the control period. Since giving a placebo gel with no nutrients to dialysis patients may raise ethical concerns, it is not feasible to use a placebo in this study. Without a placebo, there could be some subject biases. Nevertheless, with a randomized crossover design, the biases should have been lessened. Future studies are warranted to compare the efficacy of Nutri‐jelly with that of other oral nutrition supplements. The second limitation is the short duration of 7 days. Certain parameters show some trends of change but are not statistically significant. Future studies should extend the period of Nutri‐jelly intake to at least 30 days according to previous studies that show a significant impact on the nutrition status of dialysis patients (Weiner et al. 2014). Some studies demonstrated that 3 months may be required to see the significant effect of oral nutrition supplements on nutritional status and muscle mass (Rattanasompattikul et al. 2013; Liu et al. 2018). Future studies should investigate the long‐term efficacy of Nutri‐jelly on nutrition and other related parameters including total nitrogen, protein load, inflammation parameters, and muscle mass, as previously described (Castro‐Barquero et al. 2022; Stark et al. 2011). The third limitation is the small sample size (total *n* = 20 for crossover trials, *n* = 10 per group). Though the parameters with statistically significant results such as eGFR and potassium have adequate power of more than 0.8, other parameters such as albumin have inadequate power. To increase statistical power to 0.95, future studies would require total participants of at least *n* = 34 for a randomized crossover trial (*n* = 17 per group).

## 5 Implications For Clinical Practice

Nutri‐jelly is safe and may have some potential benefits as a candidate for oral nutritional supplement, especially for renal patients who need to limit fluid intake. Further studies with longer duration in a larger number of patients are warranted to evaluate the efficacy of Nutri‐jelly. Additionally, nutritional and inflammatory biomarkers should be measured.

## 6 Conclusion

The findings of this study suggest that consuming two cups per day of Nutri‐jelly (100 g/ cup) for 7 days is safe in hemodialysis patients. No adverse signs and symptoms have been observed. Nutri‐jelly intake might improve some renal‐related parameters including a significant increase in eGFR during the jelly intake period, and significantly better control of potassium level and pre‐HD diastolic blood pressure, compared to those of without‐jelly period. Furthermore, there was a trend in decreasing BUN value and phosphorus level, but not statistically significant. Due to the short duration of 7 days, this study does not show the efficacy of Nutri‐jelly on nutrition status. Further studies with longer time and larger groups of patients are warranted. Nutri‐jelly is safe and may have some potential benefits warranting further studies. It is a candidate for nutritional supplements, especially for renal patients who shall limit fluid intake.

## Author Contributions

**Janjiraporn Kitiya:** conceptualization (equal), data curation (lead), formal analysis (lead), investigation (lead), methodology (lead), writing – original draft (lead). **Thun Chantaramungkorn:** conceptualization (supporting), investigation (supporting). **Apinya Pantoe:** data curation (supporting), investigation (supporting). **Chaowanee Chupeerach:** conceptualization (supporting), methodology (supporting), supervision (supporting). **Dunyaporn Trachootham:** conceptualization (lead), formal analysis (supporting), methodology (lead), supervision (lead), writing – review and editing (lead).

## Conflicts of Interest

The authors declare no conflicts of interest.

## Supporting information


Table S1–S4

## Data Availability

No individual data are available due to ethical restrictions.
