# Supplementary material for: Short‐Term Safety of Nutri‐Jelly in Adults Undergoing Hemodialysis
Source: Food Sci Nutr. 2024 Nov 12;12(12):10507–16. doi: 10.1002/fsn3.4578 (PMC11666814; doi:10.1002/fsn3.4578)
Supplement: Supplementary file 1 — Table S1–S4 [file FSN3-12-10507-s001.docx]

**Supplementary materials**

**Short-term Safety of Nutri-Jelly in Adults Undergoing Hemodialysis**

**Table S1: Nutrition values of Nutri-jelly mango flavored per 100 g**

| **Nutrient** | **Nutri-jelly**  **Mango flavor** | **% Thai RDI** |
| --- | --- | --- |
| **Energy** | 130 kcal |  |
| **Total carbohydrate** | 18g | 6% |
| **Protein** | 5g |  |
| **Total fat** | 4g | 6% |
| **Saturated fat** | 2g | 10% |
| **Cholesterol** | 15mg | 5% |
| **Dietary fiber** | 2g | 8% |
| **Sodium** | 45mg | 2% |
| **Vitamin A** | 64 µg RAE | 8% |
| **Vitamin B 2** | 0.17 mg | 10% |
| **Calcium** | 120 mg | 15% |
| **Iron** | 0.3 mg | 2% |
| **Phosphorus** | 86.8 mg | 10.8% |
| **Potassium** | 175 mg | 0.05% |
| **Sodium** | 46.9 mg | 2.3% |

**Table S2: Compliance with Nutri-PEITC jelly intake**

| **Date of jelly consumption** | **1^st^day** | **2^nd^day** | **3^rd^day** | **4^th^day** | **5^th^ day** | **6^th^ day** | **7^th^ day** |
| --- | --- | --- | --- | --- | --- | --- | --- |
| **Finish all** | 20 | 20 | 20 | 20 | 20 | 20 | 20 |
| **Not finish all** | 0 | 0 | 0 | 0 | 0 | 0 | 0 |

The table shows the number of participants consuming Nutri Jelly as specified.

**Table S3: Summary of adverse symptoms**

| **Adverse Events** | **Without jelly (N = 20)** | **With jelly (N = 20)** |
| --- | --- | --- |
| Nausea | 0 | 0 |
| vomiting | 0 | 0 |
| Diarrhea | 0 | 0 |
| Itching | 0 | 0 |
| Fatigue | 0 | 0 |
| Edema | 0 | 0 |

Data are shown as the number of participants with adverse symptoms.

**Table S4 Comparison of energy and nutrient intakes of other food besides Nutri-jelly on hemodialysis day, non-hemodialysis day, and all days during with and without Nutri-Jelly periods**

|  | **Hemodialysis day** | | | **Non-hemodialysis day** | | | | **Average of all days** | | | |  |  |  |  |
| --- | --- | --- | --- | --- | --- | --- | --- | --- | --- | --- | --- | --- | --- | --- | --- |
|  | **Without jelly period** | **With jelly period** | **p-value** | **Without jelly period** | **With jelly period** | | **p-value** | **Without jelly period** | **With jelly period** | **p-value** | |  |  |  |  |
| **Energy (kcal)** | 1244.87 ± 756.56 | 1225.77 ± 478.02 | 0.78 | 1265.41 ± 611.1 | 1356.3 ± 426.54 | | 0.37 | 1255.14 ± 625.73 | 1286.25 ± 433.06 | 0.62 | |  |  |  |  |
| **Protein**  **(g)** | 67.21 ± 42.93 | 56.56 ± 24.64 | 0.62 | 61.54 ± 24.92 | 68.95 ± 30.46 | | 0.41 | 64.37 ± 30.85 | 62.47 ± 24.47 | 0.82 | |  |  |  |  |
| **Potassium (mg)** | 1262.97 ± 1106.11 | 1086.01 ± 849.06 | 0.62 | 983.18 ± 474.67 | 1149.76 ± 540.75 | | 0.42 | 1123.07 ± 677.08 | 1116.08 ± 533.62 | 0.65 | |  |  |  |  |
| **Phosphorus (mg)** | 501.35 ± 308.00 | 520.26 ± 315.38 | 0.98 | 522.87 ± 216.44 | 584.19 ± 297.59 | | 0.77 | 512.11 ± 214.64 | 549.73 ± 236.44 | 0.65 | |  |  |  |  |
| **Sodium**  **(mg)** | 1671.811 ± 1130.66 | 1696.88 ± 761.36 | 0.59 | 2118.10 ± 1412.27 | 2569.48 ± 1488.22 | | 0.27 | 2093.47 ± 1037.32 | 2133.26 ± 975.97 | 0.71 | |  |  |  |  |
| **Fat**  **(g)** | 48.45 ± 57.71 | 42.95 ± 16.6 | 0.79 | 49.12 ± 32.85 | 51.41 ± 28.24 | | 0.21 | 48.78 ± 39.46 | 47.02 ± 20.01 | 0.54 | |  |  |  |  |
| **Carbohydrate (g)** | 134.99 ± 54.79 | 153.25 ± 69.01 | 0.22 | 144.29 ± 70.69 | 154.45 ± 37.92 | | 0.62 | 139.64 ± 57.94 | 153.28 ± 48.98 | 0.33 | |  |  |  |  |
|  | | |  | | |  |  | |  | |  |  |  |  |  |

This table shows the mean ± SD of average intakes in 20 patients during the specified periods. P-values were obtained from Mann-Whitney tests.
